# Loot

**Published:** 1872-10

**Authors:** 


### Loot.

***Of Drains, Smells, and Fevers.***

The free ventilation that has recently been given to the unsavory subject of sewers and drains, in connection with outbreaks of typhoid fever, will, it is to be hoped, lead to practical ventilation of the sewers themselves, so that the noxious gases pent therein, and panting for escape, will be accommodated in this respect in a way as little prejudicial as possible to the health of the community. Time was when main drainage was unknown; houses were drained into cess-pits, and from time to time a functionary known as a "nightman," visited the premises in the dead of night, and bucketed out all the collected filth into his cart, to be carried away and used as manure. It did not appear that these nightmen were specially liable to fever or other disease in consequence of their avocation; indeed, the belief among some was that the occupation was rather healthy than otherwise, perhaps in consequence of the stimulating ammoniacal odors so freely inhaled.

However, we have now changed all this; at least, in most large towns; and houses are put in connection with an almost endless vista of long drains, which, while rather carriers than reservoirs of sewerage matter, are always full of most poisonous gas.

How many outbreaks of typhoid fever, especially in windy watering-places, have been caused by the current of wind up these sewers, forcing the gases into houses, it is needless to say. One well-known instance we can easily call to mind, where, at the suggestion of a well-known engineer, the introduction of ventilators protected by charcoal was a prompt means of thoroughly arresting further outbreak of typhoid in the place.

A consideration of these matters of experience will show that flushing the sewers of any house or district, at a moderate elevation above the trunk sewer, must be most dangerous, for the reason that, as the torrent of water rushes along, it displaces the gases that are collected in the sewer, and these, rising upwards, may be driven into a dwelling-house with all the force that is exercised by the pressure of the current of water.

It is, I presume, to guard against this very evil that Mr. Rawlinson has suggested the simple but most rational expedient of having the lid of every water-closet perforated with a hole just over the handle, so that this may be pulled up while the lid is closed, and so the stinking gases, displaced by the water-flow, cannot enter the house.

It would be out of place here to enter into the arrangements by which house-drains can be safely flushed. The principle to be kept in view is, that the gases rise up as the water goes down; and such arrangements in the way of ventilation must be made as will secure for these gases a free outlet into the open air. The old-fashioned cess-pool of stagnant filth may have its objections; but practice tends to prove that sewage in active motion may be more dangerous to the public health than the same matter stagnant and at rest.—*The Doctor.*

### *Management of Cerebro-Spinal Meningitis.*

J. Maclay Armstrong, M.D., Edwardsville, Ill. (*Med. and Surg. Reporter*), writes that after the relief of the brain symptoms, spotted-fever should be treated as any ordinary case of sub-acute rheumatism. He uses the following mixtures:

R. Tinct. xanthoxylum, tinct. cimicifuga rac., tinct. guaiacum, aa oz. ij.; tinct. colchicum rad., oz. j.; potas. acetas, dr. j.; syr. simplex, spts. vin. gallici, aa oz. ij. M. Sig. A dessert-spoonful every four hours.

R. Quin. sulph., gr. xj.; tinct. ferri chlor., dr. ij.; syr. simplex, aquæ menthe pip., aa oz. j. M. Sig. A dessert-spoonful three times a day, with Dover's powders at night, if necessary.

He has had under his care thirty-two cases of this disease; three proved fatal within forty-eight hours; the remainder recovered. He is also convinced that this disease should be known as cerebro-spinal rheumatosis.—*Med. Record.*

